# Spreadsheet Tools for Quantifying Seepage Flux Across the GW-SW Interface

**DOI:** 10.1029/2019wr026232

**Published:** 2021

**Authors:** R. G. Ford, B. K. Lien, S. D. Acree, R. R. Ross

**Affiliations:** 1 Office of Research and Development, USEPA, Cincinnati, OH, USA,; 2 Office of Research and Development, USEPA, Ada, OK, USA,

## Abstract

Identifying the spatial distribution and magnitude of seepage flux across the groundwater-surface water (GW-SW) interface is critical for assessing potential impairments and restoration alternatives for water bodies adjacent to sites with groundwater contamination. Measurement of the vertical distribution and time-varying characteristics of temperature in sediments provides an indirect way to map out spatial and temporal patterns of seepage flux into surface water. Two spreadsheet-based calculation tools are introduced that implement four one-dimensional analytical solutions to calculate the magnitude and direction of seepage flux based on measurement of steady-state vertical temperature profiles or transient diel temperature signals at two depths within sediment. Performance of these calculation tools is demonstrated for a pond receiving contaminated groundwater discharge from an adjacent landfill. Transient versus steady-state model performance is compared, and limitations of transient modelsare illustrated for a situation with unfavorable sediment characteristics and inadequate sensor spacing. The availability of a range of analytical solutions implemented within Microsoft Excel^®^ is intended to encourage practitioners to explore use of this seepage flux characterization method and develop greater insight into best practices for model selection and use.

## Plain Language Summary

Open-access spreadsheet-based calculation tools are provided to make use of sediment temperature for estimating the magnitude and direction of seepage flux between groundwater and surface water. The performance of the different models coded in these tools is illustrated for a study site influenced by varying weather conditions.

## 1. Introduction

Identifying the spatial distribution and magnitude of seepage flux into surface water is critical for assessing potential impairments and restoration alternatives for water bodies adjacent to sites with groundwater contamination (Conant, 2004; Conant et al., 2004; Duncan et al., 2007; Kalbus et al., 2007; Rønde et al., 2017; Selker et al., 2014; Zachara et al., 2016). While there are several alternative methods for characterizing water exchange across the groundwater-surface water (GW-SW) interface (Rosenberry & LaBaugh, 2008), use of temperature-based methods for reconnaissance surveys and quantitative assessments at the site scale provides cost-effective options that are relatively easy to implement (Kurylyk et al., 2019). There are currently a range of commercial devices for measuring sediment temperature at reasonable cost that provide the user with many options to acquire temperature data over scales and time periods that are needed to adequately characterize the spatial and temporal variability in seepage flux. Computer-based computational tools have recently been developed for a variety of software formats to extend use of temperature data for quantitative estimates of seepage flux direction and magnitude (Gordon et al., 2012; Irvine et al., 2015; Koch et al., 2016; Kurylyk et al., 2017; Schneidewind et al., 2016; Swanson & Cardenas, 2011; Voytek et al., 2014).

The purpose of this paper is to provide open access to implementations of a selection of analytical solutions to the one-dimensional (1-D) convective-conductive heat transport equation using the Excel^®^ software platform. The basis for development of these calculation tools and examples of their application for characterization of seepage flux at contaminated sites was previously published (U.S. EPA, 2014). Irvine (2018) has highlighted the importance of developing and sharing usable tools that facilitate use by practitioners working in the field of hydrogeology. The calculation tools introduced here are not viewed as superior to other open-access calculation tools. Rather, the intent of this work is to provide a range of calculation tools under a common format that provides flexibility in matching up calculation approaches with the types of temperature data that may be available for a given study site. It is desired that access to these analytical tools will encourage users to more fully explore potential limitations of applying a single analytical approach for real systems with characteristics that deviate from the assumed conditions underpinning the various analytical solutions. Use of these calculation tools and comparison of model output to independent assessment of system hydrology will be illustrated for a subset of data collected from a monitored study site.

## 2. Development of Workbooks

To take advantage of the range of analytical solutions that have been derived to calculate seepage flux from sediment temperature measurements, two spreadsheet-based workbooks were developed. The analytical solutions have been implemented such that positive values of seepage flux are assigned to upward vertical flow. Analytical solutions implemented in these workbooks are based on assumption of a static sediment column that is not influenced by erosion and deposition during the period of observation. As has been shown by Luce et al. (2013) and Tonina et al. (2014), sediment erosion/deposition can modify temperature sensor response by changing the contact matrix and, thus, invalidate the use of a constant thermal conductivity value. Changes in contact matrix are often abrupt events, resulting in a discontinuity in the logged temperature signal at the affected depth.

The SteadyState1DSE workbook (Data Set S1) was developed to calculate seepage flux from measurements of temperature at three depths for a single point in time. This workbook implements two different analytical solutions that rely solely on shallow sediment temperature measurements (Bredehoeft & Papadopulos, 1965) or on a combination of two shallow sediment temperatures and a reference temperature from the local groundwater aquifer (Schmidt et al., 2007). The analytical solution developed in Schmidt et al. (2007) is valid only for upward seepage flux into the surface water body. When independent sources of data indicate conditions supporting recharge to the aquifer (downward vertical flux, horizontal flux toward land), preference should be given to the analytical solution developed by Bredehoeft and Papadopulos (1965). For both analytical solutions, an optimal value for seepage flux is calculated through iteration using the “Solver” add-in implemented within Excel^®^ until the mismatch between the measured and calculated temperature profiles is minimized. Implementation of the analytical solution from Bredehoeft and Papadopulos (1965) is a more limited approach than that recently published by Kurylyk et al. (2017).

The Transient1DSE workbook (Data Set S2) was developed to calculate seepage flux from measurements of shallow sediment temperature time series at two depths over a period of 24 hr. This workbook implements two different analytical solutions that calculate seepage flux from either the reduction in amplitude (Hatch et al., 2006) or a combination of the reduction in amplitude and the shift in phase of the diel signal across the two monitored depths (McCallum et al., 2012). The analytical solution from Hatch et al. (2006) was implemented in VFLUX 1.2.5 and Ex-Stream modeling tools that make use of MATLAB^®^ as the programming platform (Gordon et al., 2012; Swanson & Cardenas, 2011), and the McCallum et al. (2012) analytical solution is implemented in the most recent version of VFLUX 2.0.0 (Irvine et al., 2015; Lautz et al., 2020). Although the analytical solution by Luce et al. (2013) is also incorporated in the latest version of VFLUX 2.0.0, it is not implemented in the Transient1DSE workbook (Data Set S2) due to its similarity in calculation output with the solution derived by McCallum et al. (2012) for situations in which sediment erosion or deposition does not occur (Rau et al., 2015). Since it is designed to implement calculations for single 24-hr periods, the Transient1DSE (Data Set S2) workbook is geared more toward screening analysis of data sets collected over a period of several days. An important feature of all published models is the approach used to extract the diel temperature signal at each sediment depth.

Both workbooks contain a series of individual worksheets to help guide the user through use of the optional analytical models, along with guidance on selection of appropriate model parameters that define properties of the sediment depth interval over which temperature was monitored. The first worksheet, labeled “Read Me,” provides reference to the specific articles with published analytical solutions that are implemented in later worksheets, along with instructions on how to activate the Solver Add-in within Excel^®^. (Note that the workbook file must have an “xlsm” extension for implementation of macros by Excel^®^.) Included on the first worksheet is the password for accessing the underlying programming code that is implemented during model calculations. The second worksheet, labeled “List of Symbols,” identifies and defines all symbols as used in the various analytical solutions. The third worksheet, labeled “Equations,” lists the specific equations that are solved within the worksheets for calculation of seepage flux. The fourth worksheet, labeled “Tutorial”, includes a step-by-step tutorial with illustrative examples for use of the calculation worksheets. The fifth and sixth worksheets in both workbooks implement calculation of seepage flux and provide a graphical interface by which the user can input sediment temperature data and other required model input parameters. The seventh worksheet, labeled “Test Data”, provides example sediment temperature data sets and model input parameters, along with the resultant model output values, to allow the user to confirm proper operation of the calculation worksheets. The eighth worksheet, labeled “Parameter Reference”, includes a critical review of recent literature to provide a realistic range of appropriate values for model input parameters that describe various properties of the sediment depth interval. Citations to referenced research are provided, so the user can review specifics on how the reported parameter values were determined and the characteristics of the study site. If there is uncertainty about use of default input values or suggested alternatives in the “Parameter Reference” worksheet, it is recommended that the user conduct sensitivity analyses to assess the importance of expending additional effort to collect site-specific measurements of sediment or fluid properties.

## 3. Using the Workbooks

Selecting an appropriate model is governed, in part, by characteristics of the temperature data. For example, surface water systems in which a diel signal does not propagate with depth in sediments prevent use of the transient models. These workbooks are designed to calculate seepage flux for a single, point-in-time event or a 24-hr period. For convenience in the following discussion, reference to a specific analytical solution is based on the name of the first author from the source publication.

For the SteadyState1DSE workbook (Data Set S1), it is advised that the user input temperature data collected at similar daily times. For the “Bredehoeft” and “Schmidt” models, sediment temperature data from multiple depths is typically collected through use of a multidepth sensor probe (Anibas et al., 2016; DeWeese et al., 2017; Naranjo & Turcotte, 2015). The “Bredehoeft” model calculation approach implemented in this workbook is based on measurement of sediment temperature from three depths, which is the minimum requirement for the calculation. There may be an advantage to use of more than three depths with higher-resolution spacing to constrain the shape of the temperature profile. Users that wish to explore this possibility can access the calculation tool published by Kurylyk et al. (2017). The “Schmidt” model requires collection of a regional groundwater reference temperature, which is typically less susceptible to time-dependent variations. However, it is advisable to use a groundwater temperature that is representative of the season during which sediment temperature is measured. Both analytical worksheets are designed with built-in prompts to warn the user of problems when entered data are incompatible with the analytical solution. An example includes a warning pop-up message that appears when temperatures do not decrease or increase sequentially with depth. If this situation occurs, the flux calculation is terminated. A temperature profile in which an intermediate depth is either higher or lower than both the upper and lower bounding temperatures indicates the sediment interval is likely not at steady state. A common cause for this situation may include periods of thermal turnover within shallow sediments during seasonal transitions.

For the Transient1DSE workbook (Data Set S2), the success of the seepage flux calculation is dependent on the ability to distinguish changes in the diel signal amplitude and phase with depth in the sediment. As depth in the sediment increases, the amplitude of the temperature variation will decrease, and there will be a delay in the timing of the peak amplitude. There are several factors that can limit successful extraction of the diel component from the total signal, some of which derive from natural perturbations or characteristics of the sediment interval that is monitored. Other factors can be controlled during the data acquisition process through selection of temperature sensors with higher accuracy and/or precision, as well as the depth of sensor placement.

Fuggle (1971) has demonstrated that daily air temperature variation can be described by the harmonic function, which is based on one or multiple sinusoidal components with different frequencies. Herein, we refer to the least squares fitting of a fourth-order harmonic function to raw temperature data as “harmonic regression”, as shown in the “Equations” worksheet in the Transient1DSE workbook (Data Set S2). This is an adaptation of the original temperature signal fitting approaches used by Keery et al. (2007) and Swanson and Cardenas (2011). Harmonic regression is implemented using the Excel^®^ Solver add-in to simultaneously fit four harmonic functions with cyclic periods of 24, 12, 8, and 6 hr, corresponding to 1, 2, 3, and 4 sinusoidal cycles per 24-hr period. Natural temperature variations are typically dominated by 24-hr period cycles, driven by the daily rotation of the Earth. Higher-frequency, interval harmonics with periods of 12, 8, and 6 hr may also be present in the temperature signal (Gordon et al., 2012; Smith, 1983). This fitting approach circumvents loss of usable data due to truncation of data records imposed by Fourier transform analysis (Irvine et al., 2017; Keery et al., 2007). The Transient1DSE user is constrained to analysis of temperature records that include at least 12 sequential measurements across a 24-hr period to help insure successful fit of the sinusoidal components. Temperature signals for which the diel component is not dominant should not be used in the seepage flux calculation, since this is an indication the system is influenced by external factors other than the daily temperature cycle.

To assist the user in diagnosing this potential problem, warning messages are triggered when the quality of the harmonic regression fit falls below certain thresholds. Two criteria are implemented within this workbook: (1) warning of a marginal fit to either or both raw temperature signals when the value of *R*^2^ from the harmonic regression falls below 0.95, and (2) termination of the seepage flux calculation when the harmonic regression fit returns a value of *R*^2^ below 0.9 for either or both raw temperature signals. These criteria are applied to the combined, fourth-order harmonic regression. An additional criterion terminates the seepage flux calculation for situations when the fourth-order harmonic regression *R*^2^ is greater than 0.9 but the fit quality of the first harmonic (24-hr period) to the total signal has *R*^2^ less than 0.5. The choice of these threshold criteria is empirical, based on application of the harmonic regression approach to multiple data sets under a range of hydrologic environments. Experience has shown that relaxing these criteria results in use of data that are influenced by factors other than the Earth’s rotation. For these cases, the day-night temperature cycle is no longer the primary external thermal signal that underlies application of the derived models.

The workbook automatically shows plots of the raw temperature data along with the resultant fit of the harmonic regression (first, diel harmonic and combined harmonics) for both sensor depths, so the user can visually assess the significance of the regression *R*^2^ value. Plots of the fitted harmonic regressions are shown for situations where calculation of a value for seepage flux is terminated due to poor fit quality. For cases where the seepage flux calculation is terminated using the Transient1DSE workbook (Data Set S2), it may still be possible to implement the calculation using the SteadyState1DSE workbook (Data Set S1). In the following section, use and limitations of the various analytical solutions implemented in these workbooks will be illustrated.

## 4. Illustration of Workbook Calculations

A long-term study site will be used to illustrate application of the various models and compare output between steady-state and transient models for a given set of sediment temperature data. All data were collected within a cove that is part of a larger, flow-through lake (Plow Shop Pond) adjacent to Shepley’s Hill Landfill in Devens, Massachusetts. These data are documented within the supporting information file (Table S1). Geologic units near the Shepley’s Hill Landfill and Plow Shop Pond consist primarily of glacial deposits overlying an irregular bedrock surface of metasedimentary rocks, gneisses, and granites. The overburden glacial deposits beneath the landfill are primarily composed of well-graded to poorly graded sands with silts and gravel and, in some areas, peat from previously existing wetlands. A discontinuous layer of till is present at the base of the sands directly overlying bedrock.

**Figure 1.**
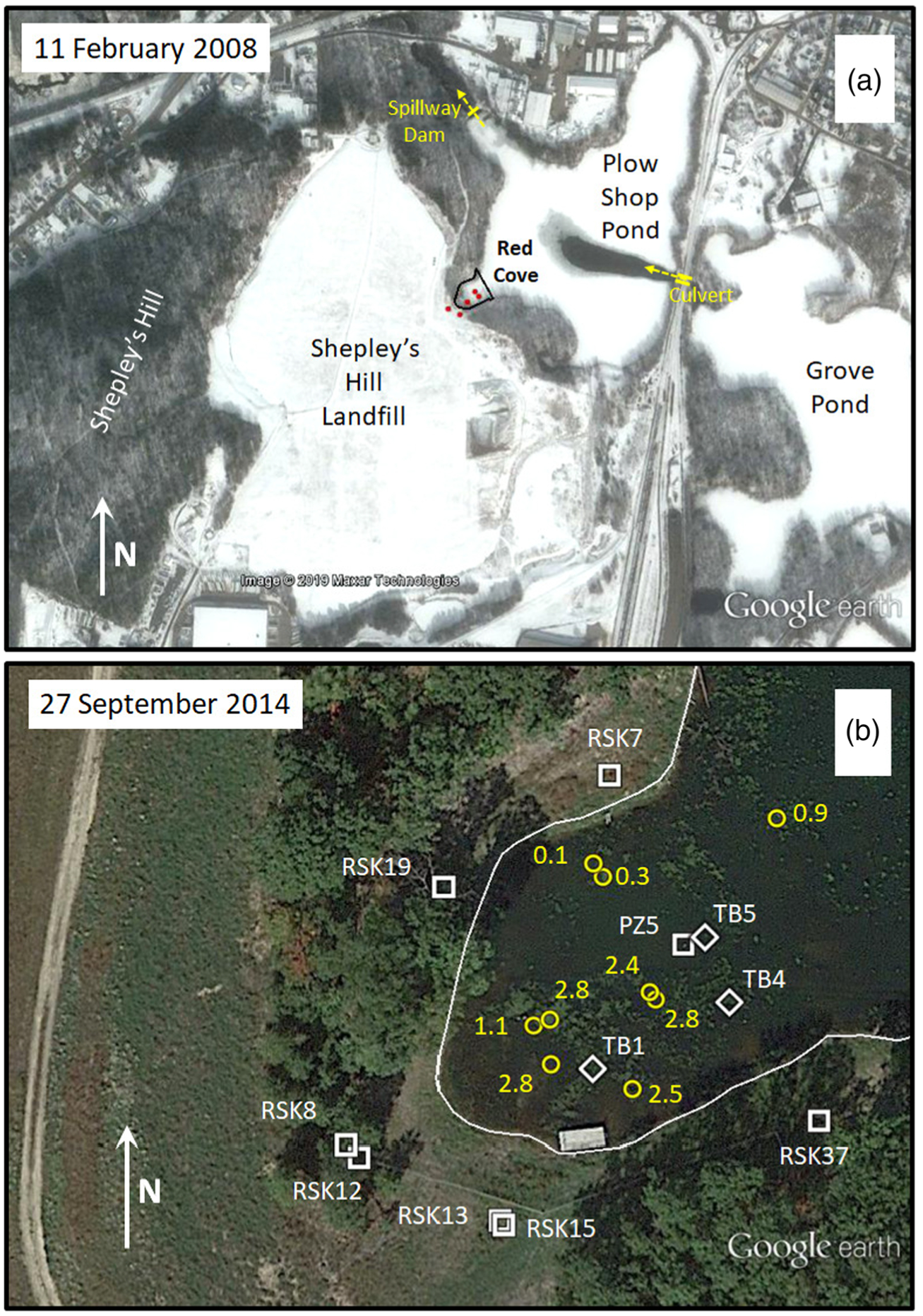
(a) Aerial view of landscape features surrounding Red Cove (black outline). Yellow arrows highlight the flow inlet and outlet for Plow Shop Pond. (b) Close-up view of Red Cove (white outline) showing the locations of piezometer clusters (squares) on land around the cove perimeter, along with sediment temperature profile locations (diamonds) and shallow piezometer (square) within the cove. Also shown are locations of manual seepage meter measurements (yellow circles) conducted during April 2007 and June 2008; seepage flux values (cm day^−1^) are posted next to the symbol.

Plow Shop Pond, including Red Cove, is bounded on the west by the landfill and, further to the west, Shepley’s Hill, a bedrock topographic high. Plow Shop Pond receives surface water flow from Grove Pond to the east and discharges into a tributary to Nonacoicus Brook at the northwest end of the pond (Figure 1a). Water level in Plow Shop Pond is regulated by a spillway dam where water from the pond discharges into the tributary. In general, groundwater discharges to the southern portion of Plow Shop Pond, including Red Cove, from areas south and southwest of the pond. This situation reverses beneath the northern half of the pond with surface water generally providing recharge to the aquifer. The cove is separated from the main flow of water through the lake and is not significantly affected by currents. Information on the conditions within the cove, as well as specifics on the monitoring network within the cove and the adjacent groundwater system, has been previously published (Ford et al., 2011; U.S. EPA, 2008, 2009).

For this illustration, data collected during August 2014 are used to compare calculated direction and magnitude of seepage flux using the four analytical solutions coded into the steady-state and transient workbooks. The physical condition of the cove during the study period was consistent with the aerial image shown in Figure 1b. Data analyzed in this article were collected from piezometers installed within the aquifer adjacent to the cove at the groundwater table (RSK12, RSK15) and above the bedrock surface (RSK8, RSK13), temperature profilers installed within cove sediments (TB1, TB4, TB5), and a shallow piezometer installed in the middle of the cove (PZ5). A period of 13 days of data was selected, centered around an isolated, 1-day storm event. Details on the characteristics of these monitoring locations and data collection procedures are provided in the supporting information.

**Figure 2.**
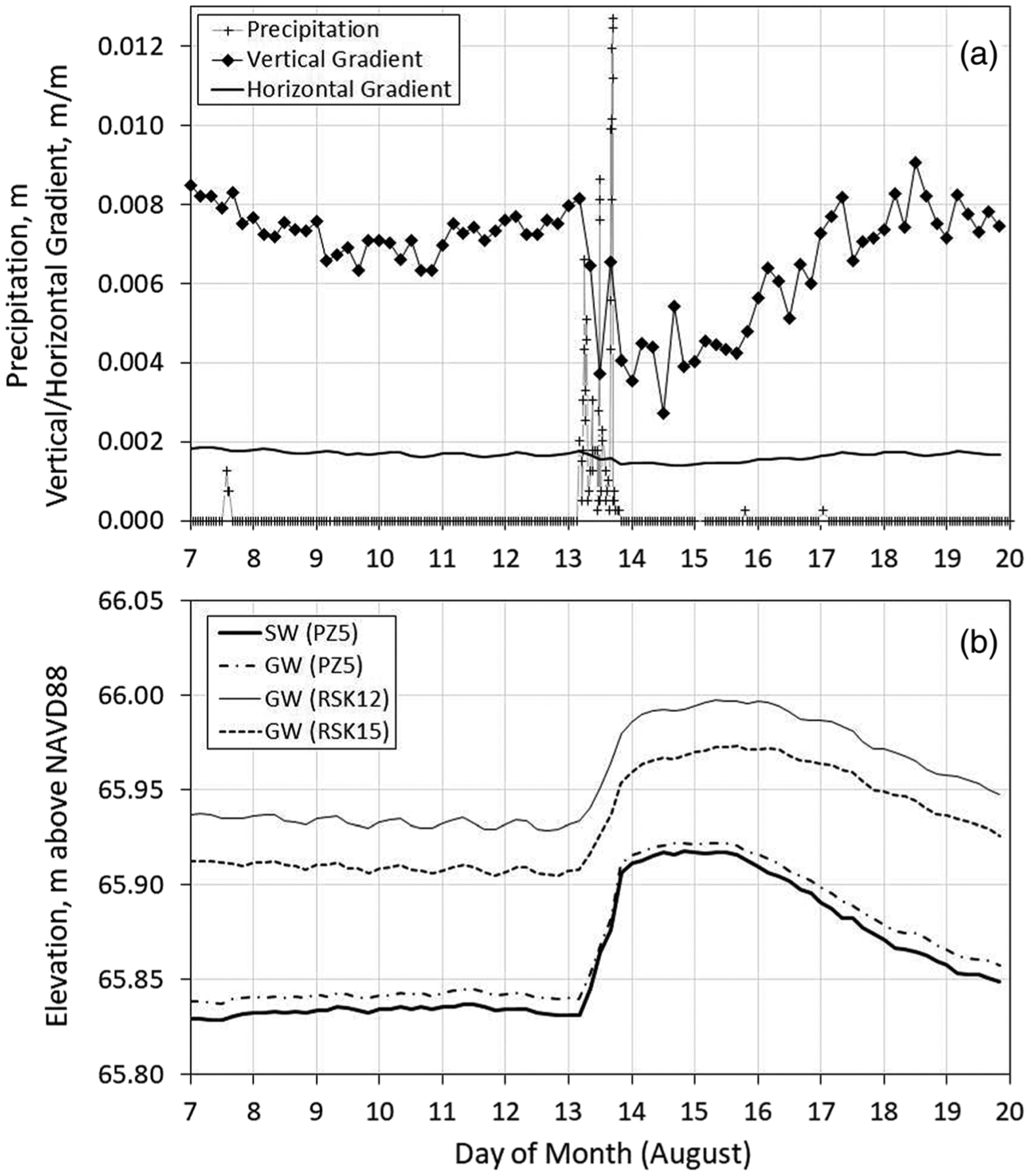
(a) Trends in precipitation during the period of seepage flux analysis from a nearby airport and response in horizontal (toward cove) and vertical (upward) gradient between groundwater and surface water within the cove based on groundwater head measurements at RSK12, RSK15, and PZ5. (b) Elevation trends for the water surface within the cove at PZ5 and the groundwater potentiometric surface in the aquifer at piezometers Locations PZ5, RSK12, and RSK15.

A summary of factors influencing local hydrologic conditions at the study site are illustrated in Figure 2. In Figure 2a, hourly precipitation data from a local weather station are shown along with measured horizontal and vertical gradients for groundwater flow toward and into the cove (Table S1 [Table S1.1]). During this time, there was an isolated period of rain during 13 August resulting in an accumulated rainfall of 0.19 m. As shown in Figure 2b, the elevation of surface water within the cove rose quickly in response to this precipitation event, accompanied by increases in the groundwater potentiometric surface elevation underneath the cove. As a result, there was a nearly instantaneous change in the vertical gradient, which slowly returned to prestorm conditions within a few days. While less dramatic, this same change was observed in the measured horizontal gradient governing flow to the cove. In both cases, the rise in surface water elevation decreased the driving force for groundwater flow toward and into the cove. The isolated precipitation event on 13 August disturbed the daily temperature fluctuation within surface water, which appeared to be reestablished on the following day (Supporting Information S1 [Figure S1] and Table S1 [Table S1.2]).

Observations of sediment temperature at three locations within the cove are reported in Table S1 (Table S1.3). The thermal conductivity of sediment at these locations was measured using a single needle heat pulse sensor referenced to a standard material as a performance check (supporting information; Yi et al., 2009). The measured values for five sediment cores capture the range of measured thermal conductivity throughout the cove (Table S1 [Table S1.4]). The depositional setting within the cove is characterized by a patchwork of biologically productive areas with accumulation of organic matter at the surface (Figure 1b) and less-productive areas dominated by the underlying sandy-textured sediment that is consistent with the surrounding shallow aquifer solids. For Location TB1, sediment had a relatively uniform thermal conductivity (1.50 ± 0.30 W m^−1^°C^−1^; Table S1 [Table S1.4]) consistent with its sandy texture. In contrast, Locations TB4 and TB5 had greater accumulation of organic matter, resulting in lower thermal conductivity (TB4N0.59 ± 0.03 W m^−1^°C^−1^; TB4S 1.17 ± 0.44 W m^−1^°C^−1^; TB5–1 0.73 ± 0.44 W m^−1^°C^−1^; TB5–2 0.55 ± 0.01 W m^−1^°C^−1^; Table S1 [Table S1.4]). For Location TB1, a diel variation in temperature was observed at the sediment surface and 10 cm below the sediment surface. For Locations TB4 and TB5, there was no evidence of a diel signal for temperature sensors below the sediment surface. The lack of an oscillating temperature for two monitored sediment depths limits the type of seepage flux model that can be applied at Locations TB4 and TB5.

Calculated seepage flux from the different solutions implemented in the SteadyState1DSE (Data Set S1) and Transient1DSE workbooks (Data Set S2) are summarized in Table 1. Positive values of seepage flux correspond to discharge or upward vertical flow into the cove. As a point of reference, the measured vertical gradient at PZ5 is also shown. Estimates of horizontal Darcy flux (Beljin et al., 2011) for the adjacent aquifer and vertical Darcy flux at shallow piezometer PZ5 are provided in Tables S7 and S8.

Only Location TB1 displayed sediment temperature with diel fluctuations at two monitored depths in sediment. Both the “McCallum” and “Hatch” models were used to calculate seepage flux for sediment temperature data at 0- and 10-cm depth within sediment. For the “McCallum” model, the magnitude and direction of calculated seepage flux varied widely between −15 cm day^−1^ (downward) and +15 cm^−1^ (upward). Poor fits to the diel temperature signals caused failure of the model for two days (13 and 15 August), including the day of the precipitation event (harmonic regression fit *R*^2^ < 0.9 for 0-cm depth). Suboptimal harmonic regression fits were observed at the 10-cm depth for additional days (*R*^2^ < 0.95 on 10, 11, 14, and 16 August). The occurrence of unsuccessful or poor harmonic regression fits did not appear to be limited by the resolution (0.0625°C) of the temperature sensing device (Supporting Information S1 [Figure S5]). Rather, the root cause of this outcome was the diminishing dominance of the diel component of the daily temperature cycle. As has been noted in the literature, perturbation to diel temperature cycles may be attributed to physical phenomena within the water body that push the sediment temperature profile away from a thermal state required by the assumed conditions of the analytical model (Chen et al., 2018; McCallum et al., 2012; Rau et al., 2015; Sebok et al., 2017). Likewise, the presence of a shallow sediment layer with relatively low thermal conductivity may limit the ability of the sediments to maintain true thermal equilibrium. For example, the lack of diel signal propagation at Locations TB4 and TB5 may be attributed, in part, to observed lower thermal conductivity for shallow sediments. This highlights the value of assessing the magnitude and spatial variability of sediment thermal conductivity for locations selected for acquisition of temperature profile data (Duque et al., 2016; Sebok & Muller, 2019; Sebok et al., 2015).

Calculated seepage flux was also examined using the “Hatch” model in order to see if poor resolution of phase shift within the harmonic regression caused the highly variable output from the “McCallum” model. The “Hatch” model was implemented with two options for thermal conductivity that aligned with either the shallowest, 5-cm sediment interval (0.95 W m^−1^°C^−1^) or the average of the values for the 5- and 10-cm intervals (1.33 W m^−1^°C^−1^). This resulted in calculated seepage flux values with a range between −8 and +25 cm day^−1^ and between −1 and +40 cm^−1^, respectively. Output from the “Hatch” model followed the same daily trend as shown for output from the “McCallum” model. Ultimately, the seepage flux values calculated using the transient models were generally inconsistent with the observed gradients supporting groundwater flow into the cove. Estimation of the limit of adequate downward vertical propagation of the diel signal at Location TB1 was examined based on equations for calculating “extinction depth” (Briggs et al., 2014; Irvine et al., 2017) or “diurnal damping depth” (Luce et al., 2013). This analysis was conducted for dates on which the harmonic regression fit *R*^2^ was greater than 0.9. Calculated “extinction depth” based on a sensor resolution of 0.0625°C ranged between 1.5 and 7.0 cm for values of seepage flux between 1.5 and 5.0 cm day^−1^. Likewise, calculated “diurnal damping depth” based on sensor spacing and calculated amplitude ratio and phase shift for diel temperature signals ranged between 4.5 and 9.0 cm. A final check was conducted using an independent data set (McCobb et al., 2018) to confirm that the analytical solutions were properly implemented in the Transient1DSE workbook (Table S1 [Table S1.6]). These results indicate that the combination of sediment properties and sensor spacing used for the Red Cove study site were unsuitable for application of transient models at Location TB1.

Use of the steady-state models that rely on temperature-depth profile data were subsequently examined. Sediment thermal conductivity is a required input parameter for both models implemented in the SteadyState1DSE workbook (Table S1). For application of model calculations for different vertical intervals, the average of measured sediment thermal conductivity values from the corresponding depth intervals of sediment cores was used (Table S1 [Table S1.4]). Also, the average of the daily temperature signature for each depth was used as input into the calculation models. The results of these calculations are presented in Table 1 for Locations TB1, TB4, and TB5. For implementation of the “Bredehoeft” analytical solution, combinations of the top three or bottom three temperature sensors were used. For Location TB1, both depth profiles displayed upward seepage flux values that diminished in magnitude in response to the rainfall event followed by a gradual return to prestorm values after several days. The same general trend in upward seepage flux was observed for Locations TB4 and TB5. For implementation of the “Schmidt” analytical solution, daily average sediment temperatures were used in combination with the average temperature of groundwater (RSK8, RSK13) for the entire time period (12.06°C) to calculate seepage flux. For Location TB1, temperature sensors placed at 10- and 52-cm depths were used, while sensors at depths of 10 and 60 cm were used for Locations TB4 and TB5. Again, the magnitude of calculated, upward seepage flux decreased following the rainfall event. It should be noted that the calculated seepage flux for the steady-state models scales linearly with sediment thermal conductivity, which varied between 0.5 and 2.0 W m^−1^°C^−1^ for the three sediment cores adjacent to these locations. For the “Bredehoeft” model, adjustment of the input thermal conductivity to the smallest measured value for all sediment cores resulted in calculated seepage flux less than 10 cm day^−1^ for both depth intervals at both locations.

## 5. Conclusions

In general, the steady-state models implemented in the two workbooks generated time-dependent patterns in seepage flux that reproduced expected behavior from response of the hydrologic system to perturbation from the isolated rain event. Comparison between seepage flux derived from sediment temperature profile measurements and to measured vertical hydraulic gradient at PZ5 showed a consistent pattern of water movement from the shallow aquifer into the surface water body. As shown for the data set examined in this article, the steady-state models appeared to perform better than the transient models for this study site. Supporting calculations examining the depth of penetration for a usable diel signal suggested that the combination of sediment thermal conductivity, sensor spacing, and an upward seepage flux may limit use of the transient models for this study site. This is not unexpected based on prior research that has highlighted potential limitations of transient 1-D models in areas dominated by upward flux (Briggs et al., 2014; Kurylyk et al., 2017) and deviations from one-dimensional flow or physical perturbations that push the sediment package to a nonstationary condition (e.g., storm events). This situation is likely exacerbated for the Red Cove study area where the top sediment layer has a significant organic carbon component that impedes transmission of thermal signals (see Chen et al., 2018). One approach to address this issue might be to use shallower temperature sensors (Briggs et al., 2014; Irvine et al., 2017). Unfortunately, sediment temperature data at shallower intervals were not available at this study location to examine whether transient model output might be reconciled with other observations that supported the lack of a driving force for downward seepage flux. The results of this evaluation suggest that evaluations using multiple 1-D models can be employed to establish whether there is general consistency over a larger sediment depth interval. As demonstrated for this study, the use of alternative models may be mandated by limitations in temperature signal characteristics (e.g., lack of usable diel signal).

It is recommended that the data collection effort be designed to address not only specific technical questions for the system under study, but in addition, situations where the sediment temperature data may not adhere to the requirements of the various 1-D models. This includes deployment of temperature sensors at a range of depths targeted to facilitate use of both transient and steady-state 1-D models. Likewise, collection of supplemental data such as sediment thermal conductivity and vertical hydraulic gradient can assist in decisions for appropriate model application. Finally, use of logging temperature sensors is critical to help inform later assessments of conditions that might limit applicability of the various 1-D models. Examination of the raw temperature data may alert the analyst to periods of time that may violate the underlying assumptions of the analytical models. Use of the various 1-D models programmed into the Transient1DSE and SteadyState1DSE calculation tools, in combination with their built-in data screening criteria, provides a way to critique appropriate model application and address limitations of the monitoring network design or loss of instrumentation during the temperature monitoring period.

## Supplementary Material

Supporting Information S1

Table S1

Data Set S1

Data Set S2

## Figures and Tables

**Table 1:** Comparison of Measured Vertical Hydraulic Gradient at PZ5 to Calculated Seepage Flux Output for Daily Average Temperature Profiles (“Bredehoeft” and “Schmidt”) or Daily Temperature Trends (“McCallum” and “Hatch”) for Locations TB1, TB4, and TB5

August 2014	7	8	9	10	11	12	13	14	15	16	17	18	19
PZ5	Vertical Gradient (mm m^−1^)												
	8.1	7.4	6.9	6.7	7.3	7.5	6.1	4.1	4.4	6.0	7.3	8.0	7.6
TB1	Daily Calculated Seepage Flux (cm day^−1^)												
Bredehoeft	19.0	17.8	18.5	19.2	19.7	19.2	18.7	15.6	16.5	15.8	16.4	17.0	17.9
Schmidt	4.4	4.2	4.1	4.3	4.3	4.3	4.1	3.5	3.3	3.1	3.1	3.1	3.3
McCallum	−7.9	−4.7	11.2	13.9	13.4	−0.1	NS	−14.9	NS	−13.7	−8.1	4.3	0.6
Hatch	6.0	11.8	24.5	23.9	23.2	15.5	NS	−7.6	NS	−4.3	6.7	21.8	19.4
TB4	Daily Calculated Seepage Flux (cm day^−1^)												
Bredehoeft	2.9	2.7	2.3	2.5	3.1	2.7	2.5	2.1	1.5	1.1	1.2	1.3	1.2
Schmidt	1.2	1.2	1.1	1.1	1.1	1.2	1.1	1.1	0.9	0.9	0.8	0.8	0.8
TB5	Daily Calculated Seepage Flux (cm day^−1^)												
Bredehoeft	4.1	3.9	3.6	3.7	3.9	4.0	3.9	3.1	2.7	2.5	2.4	2.5	2.8
Schmidt	2.5	2.5	2.4	2.4	2.4	2.5	2.4	2.3	2.2	2.1	2.0	2.0	2.0

*Note*. Model input parameters and sensor configuration are provided in Tables S7 and S8. NS indicates no solution could be derived due to inadequate data quality.

## Key Points:

- Open-access spreadsheet-based calculation tools are presented for estimating vertical seepage flux from sediment temperatures
- Data from a study site is used to compare performance of various one-dimensional analytical models
- The influence of monitoring network design and precipitation events on the usability of model output is demonstrated
