# Supplementary material for: Spreadsheet Tools for Quantifying Seepage Flux Across the GW-SW Interface
Source: Water Resour Res. Author manuscript; Available in PMC 2022 Jan 1. (PMC7970483; doi:10.1029/2019wr026232)
Supplement: Supporting Information S1 [file NIHMS1673426-supplement-Supporting_Information_S1.pdf]

Water Resources Research

Supporting Information for

Spreadsheet Tools for Quantifying Seepage Flux Across the GW-SW Interface

R. G. Ford[1], B. K. Lien[1], S. D. Acree[2], and R. R. Ross[2]

[1] Office of Research and Development, USEPA, Cincinnati, Ohio, USA

[2] Office of Research and Development, USEPA, Ada, Oklahoma, USA

Contents of this file

Text S1
Text S2
Figure S1
Figure S2
Figure S3
Figure S4
Figure S5

Additional Supporting Information (Files uploaded separately)

SI_Monitoring_Data_Table S1
SI_SteadyState1DSE
SI_Transient1DSE

Introduction

Supporting information includes the procedure for making the macro-enabled Excel Workbooks functional, as well as detailed procedures for data collection, including: 1) survey of location and elevation of monitoring locations,

2) measurement of groundwater and surface water elevations, 3) measurement of sediment thermal conductivity, and 4) measurement of sediment temperature.

IMPORTANT: Both Steady1DSE.xlsx and Transient1DSE.xlsx are macro-enabled workbooks which requires the user to change the file extension of the downloaded files from ".xlsx" to ".xlsm" before they can be opened. Failing to do so will result in the following error message (example): "Excel cannot open the file "SI_SteadyState1DSE.xlsx' because the file format or file extension is not valid. Verify that the file has not been corrupted and that the file extension matches the format of the file." In order to modify the file extension of each downloaded workbook, the user needs to make sure that the file extensions are visible when viewing the list of downloaded files in the folder destination. In order to view the full filename with extension, the user needs to select "File name extensions" under the "View" tab in the Windows File Explorer view. This function is enabled when the box immediately to the left of "File name extensions" has a check mark. After enabling view of file extensions, the user should click once on the filename to highlight it, then click again to highlight and change the file extension from ".xlsx" to ".xlsm". When this is done, the following pop-up message will be received: "If you change a file name extension, the file might become unusable. Are you sure you want to change it?". The user should select "Yes", after which the file will open as a macro-enabled workbook. The file will open to the first worksheet entitled "Read Me", which has additional instructions on how to ensure that the Excel Solver® add-in is enabled for proper workbook functioning.

The underlying data that support calculations of seepage flux, as well as trends in weather and water elevations presented in Figure 2 of the main text, are documented in the Supporting Information file named "SI_Monitoring_Data_Table S1.xlsx". This workbook includes the following data types: 1) groundwater and surface water elevations and local precipitation (Table S1.1), 2) temperature records for air, surface water, groundwater and barometric pressure (Table S1.2), 3) sediment temperature data at multiple depths for three locations within the cove (Table S1.3), 4) sediment thermal conductivity data for three locations within the cove (Table S1.4), 5) summary of calculated seepage flux using different models for locations TB1 and TB4 (Table S1.5), 6) comparison of measured and calculated seepage flux for an independent dataset (Table S1.6), 7) summary of calculated seepage flux using the transient models, vertical Darcy flux calculations and horizontal Darcy flux calculations (Table S1.7), and 8) summary of calculated seepage flux using the steady-state models, vertical Darcy flux calculations and horizontal Darcy flux calculations (Table S1.8). Also included

in this file are the measured and modeled harmonic fits of temperature trends for location TB1 on three different dates (Figure S5 Values).

The following section provides graphical summaries of atmospheric conditions, surface water temperature, sediment temperature, along with calculated seepage flux using various methods. This section also includes a figure providing examples when sediment temperature was perturbed by a precipitation event that prevented use of the transient model based on diel temperature signals at two depths. Three other files are included with the Supporting Information: 1) the complete data set used to conduct seepage flux calculations (SI_Monitoring_Data_Table S1), 2) the steady-state spreadsheet calculation tool with imbedded macro-enabled codes and user instructions (SteadyState1DSE), and 3) the transient spreadsheet calculation tool with imbedded macro-enabled codes and user instructions (Transient1DSE).

Text S1.

This project was conducted under an approved Quality Assurance Project Plans G-LRPCD-0030041-QP-1-0 and G-LRPCD-0012768-QP-1-1. The hydraulics of the groundwater system adjacent to and underlying the cove was characterized and monitored through installation of a network of piezometers, as detailed in published reports (USEPA, 2008; USEPA, 2009). Five nested piezometer installations were constructed using direct-push technology and consisted of a series of individual casings screened over consecutive 1.54-meter (5-foot) intervals across the groundwater table down to the depth of refusal near the top of bedrock. The latitude, longitude, and elevations of nested piezometers installed along the shoreline were established through line-of-site and GPS survey conducted under contract to the U. S. Army. Subsequently, the latitude and longitude of the temporary piezometer installed in the middle of the cove to a depth of approximately 1.83-m (6-ft) below the sediment surface was determined using GPS and the elevation of the top of casing determined by line-of-site survey referenced to the existing surveyed network on land. A 2.54-cm (1-in) PVC pipe stilling well was attached flush to the temporary piezometer to allow concurrent measurement of the depth to surface water from a common top-of-casing elevation. Relevant piezometer location data are included in Tables S1.1 and S1.2 in a separate file (SI_Monitoring_Data_Table S1).

Water elevation in piezometers RSK12, RSK15, and PZ5 was monitored through combination of manual and automated measurements. Manual measurements were conducted on a periodic basis using a Solinst electric water level indicator attached to a laser marked flat tape with graduations every 0.003-m (0.01-ft). Unvented Solinst pressure transducers (Levelogger® Edge Model

3001) were deployed below the water surface in the piezometers and stilling well for continuous logging of total pressure within the casing. Total pressure recorded by the datalogger was corrected to water pressure by subtracting local barometric pressure monitored using a Solinst Barologger Edge Model 3001 datalogger deployed within the air space of piezometer RSK12. Corrected water pressure was converted to groundwater elevation using manual depth-to-water measurements and surveyed casing tops for the piezometers and stilling well. Logged water elevation data are provided in Table S1.1 in a separate file (SI_Monitoring_Data_Table S1).

The in-situ temperature of groundwater and saturated sediments was monitored to support calculations of seepage flux. The temperature of groundwater was continuously logged in piezometers RSK12, RSK15, RSK8, and RSK13 using Solinst Levelogger® Edge Model 3001 dataloggers. The Edge Model 3001 has reported temperature accuracy of 0.05 °C with resolution 0.003 °C. The temperature of saturated sediments was monitored at two locations within the cove using Maxim iButton Temperature Loggers (Model DS1922L), each encased within a NexSens DS9010K Underwater Housing. The DS1922L iButton has reported accuracy of 0.5 °C with 0.0625 °C resolution (11-bit setting). Each temperature logger was deployed at specific depths below the sediment surface using a custom drive rod consisting of a PVC drive shoe attached to a 2.54-cm (1-in) steel pipe. The encased iButton logger was held at its deployment depth using a 1.27-cm (0.5-in) PVC pipe inserted through the steel pipe during retrieval of the drive rod. Sediment temperature loggers deployed at zero depth were resting on the sediment surface. For a given location, the iButton loggers were deployed at individual locations within an area of approximately 0.21 m^2 (2.25 ft^2) with braided, stainless-steel wire used to tether each logger casing to a support at the water surface for retrieval at the end of deployment. Experience has shown that minimizing contact between the temperature sensor and materials exposed above the sediment or water surface minimizes potential bias from heat transfer along profiler construction materials. The loggers were programmed for use at their high-resolution (11-bit) setting. Logged temperature data are provided in Table S1.2 in a separate file (SI_Monitoring_Data_Table S1). The iButton logger deployed at the sediment surface (0-cm) at location TB5 failed during the deployment period, so seepage flux calculations could only be implemented using the remaining three deployment depths (10-, 30-, and 60-cm).

The thermal conductivity of saturated sediment for the three temperature sensor deployment locations was measured to support seepage flux calculations. Sediment cores were collected using an Aquatic Research Instruments Universal Percussion Corer with polycarbonate core barrel (6.8-cm inside diameter).

Following retrieval, the top of the core barrel was cut off to eliminate free-standing water and capped. The sealed sediment core was then laid horizontal in a shaded location and approximately 0.3-cm (0.125-in) holes were drilled in a row through the top surface of the core barrel at approximately 5-cm (2-in) intervals. Thermal conductivity was measured using either a Decagon KD2 or Decagon KD2 Pro thermal properties analyzer equipped with a KS-1 single needle probe. The probe was inserted vertically through each drilled hole into the saturated sediment such that the needle was fully submerged in sediment in a direction parallel to the cross-section of the core barrel. Duplicate and replicate measurements at three different intervals were conducted to insure repeatability of measurements (minimum of 5-minute thermal recovery time between measurements). Vegetable shortening (0.1-0.2 W m^{-1} K^{-1}; Yi et al., 2009) was used as a reference material to check accuracy of instrument response prior to and following completion of measurements for each sediment core. Sediment thermal conductivity data are provided in Table S1.3 in a separate file (SI_Monitoring_Data_Table S1).

Text S2.

The influence of changing weather conditions on surface water temperature is shown in Figure S1. Hourly records of air temperature, barometric pressure, and surface water temperature demonstrate the response of surface water temperature to the precipitation event on August 13. The isolated precipitation event disturbed the daily temperature fluctuation within surface water, which appeared to be re-established on the following day. Corresponding response in sediment temperature are shown for locations TB1 and TB4 in Figure S2. Sediment temperature also displayed a decrease in temperature in response to the isolated precipitation event. The precipitation event also disturbed the daily temperature cycle observed at temperature sensors deployed at the sediment surface for locations TB1 and TB4. Inspection of these data demonstrate that TB1 was the only location at which a diel temperature signal was clearly evident at the 10-cm deployment depth.

As a point of comparison for seepage flux estimates based on sediment temperature data, calculated Darcy flux in both the horizontal and vertical direction was estimated using water elevation data from piezometer locations RSK12, RSK15, and PZ5. The horizontal Darcy flux was determined using the 3PE calculation tool (Beljin et al., 2011) and shallow groundwater elevations within the triangular area bounded by upland piezometers screened near the water table (RSK12, RSK15) and a shallow piezometer installed within the middle of the cove (PZ5). The horizontal hydraulic conductivity of aquifer sediments across the screened intervals for RSK12 and RSK15 ranged between 20 and 31 m d^{-1}, based

on slug test measurements conducted within clusters of 1.5-meter piezometer screen intervals that spanned the saturated thickness of the aquifer (USEPA, 2008). Based on measurement of vertical gradient from shallow piezometers within the cove (0.02-0.08 m m^{-1}) and direct measurements of seepage flux during April 2007 and June 2008 (values posted in Figure 1B), the vertical hydraulic conductivity (Kv) of cove sediments was estimated to vary between 0.02-1.3 m d^{-1} with a median value of 0.3 m d^{-1}. This range of vertical hydraulic conductivity falls within that commonly observed for layered, depositional environments with organic matter accumulation (Sebok et al, 2015). The vertical Darcy flux at location PZ5 was calculated based on an estimated range of 0.3-3.0 m d^{-1} for Kv and continuous measurement of vertical hydraulic gradient derived from transducer data from shallow groundwater and overlying surface water.

The flux envelopes presented for these two measurement approaches bracket the range of measured and estimated hydraulic conductivity. Comparisons of Darcy-based flux estimates to seepage flux calculated using the 1-D transient models at location TB1 are shown in Figure S3, and comparison to seepage flux calculated using the 1-D steady-state models at locations TB1 and TB4 are shown in Figure S4. The calculated Darcy flux indicates a relatively consistent discharge into the cove that was temporarily diminished by the increased surface water levels following the August 13 precipitation event. The magnitude of the Darcy flux values is comparable to historical, direct seepage flux measurements using a seepage meter, which ranged between 0.2-2.8 cm d^{-1} within the protected area of the cove (Figure 1B; USEPA, 2009). This independent source of data indicates that seepage flux values less than 10 cm d^{-1} are characteristic of groundwater discharge into the cove.

Verification of the performance of the 1-D steady-state models in the SteadyState1DSE workbook was performed through comparative analysis using two independently published 1-D convective-conductive heat transport models for temperature profile locations TB1 and TB4. The output from these models are shown in Table S1.5, along with output from the "Bredehoeft" and "Schmidt" steady-state models from the SteadyState1DSE workbook. The 1DTempPro V2 model (Koch et al., 2016) allows for flexibility in assigning sediment characteristics to multiple layers within the modeled depth interval. For this comparison, we modeled the sediment as a two-layer system, using the average measured thermal conductivity from two depth intervals from sediment cores adjacent to TB1 and TB4 (Table S1.5 footnotes). This model was run for each daily monitoring interval with the hourly temperature values from the sensors at 0-cm and 52-cm depth as the upper and lower boundary condition, respectively. Likewise, we implemented the Flux-LM model (Kurylyk et al., 2017) using the same physical

construct for the vertical distribution of sediment thermal conductivity. The Flux-LM model implements the analytical solution from Bredehoeft and Papadopulos (1965) and generates identical magnitude and direction of seepage flux as derived from the SteadyState1DSE workbook when using a homogenous thermal conductivity value across the entire sediment interval. The output from these alternative models shows similar trends as observed for output from the SteadyState1DSE workbook. In general, 1DTempPro V2 and Flux-LM model output show higher calculated seepage flux at location TB1 versus TB4, and these models show a diminished seepage flux in response to the August 13 precipitation event. Comparisons to independently published versions of the transient analytical solutions (e.g., VFLUX and Ex-Stream) could not be undertaken, since we did not have access to the required commercial software. However, it was possible to verify the "Hatch" transient solution in the Transient1DSE workbook produced seepage flux estimates comparable to those generated by VFLUX for the sediment temperature data set published by McCobb et al. (2018; see Table S1.6).

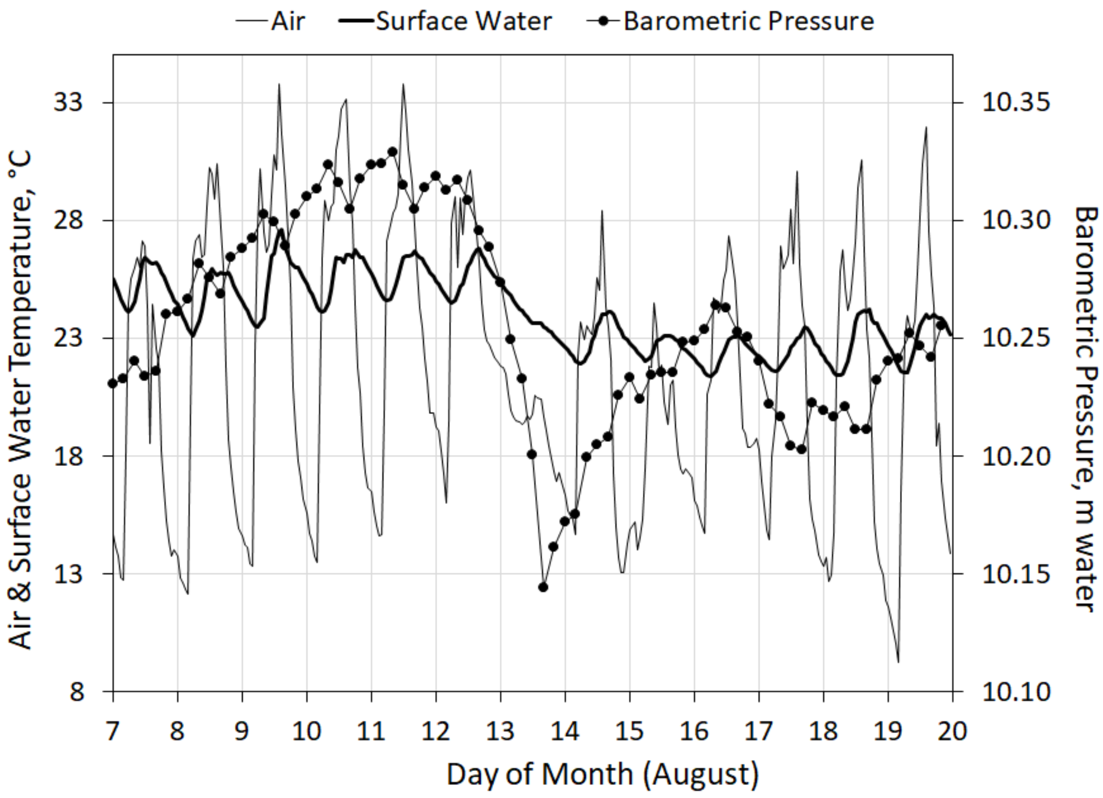

Figure S1. Trend in local air temperature, surface water temperature, and barometric pressure during the period of seepage flux analysis.

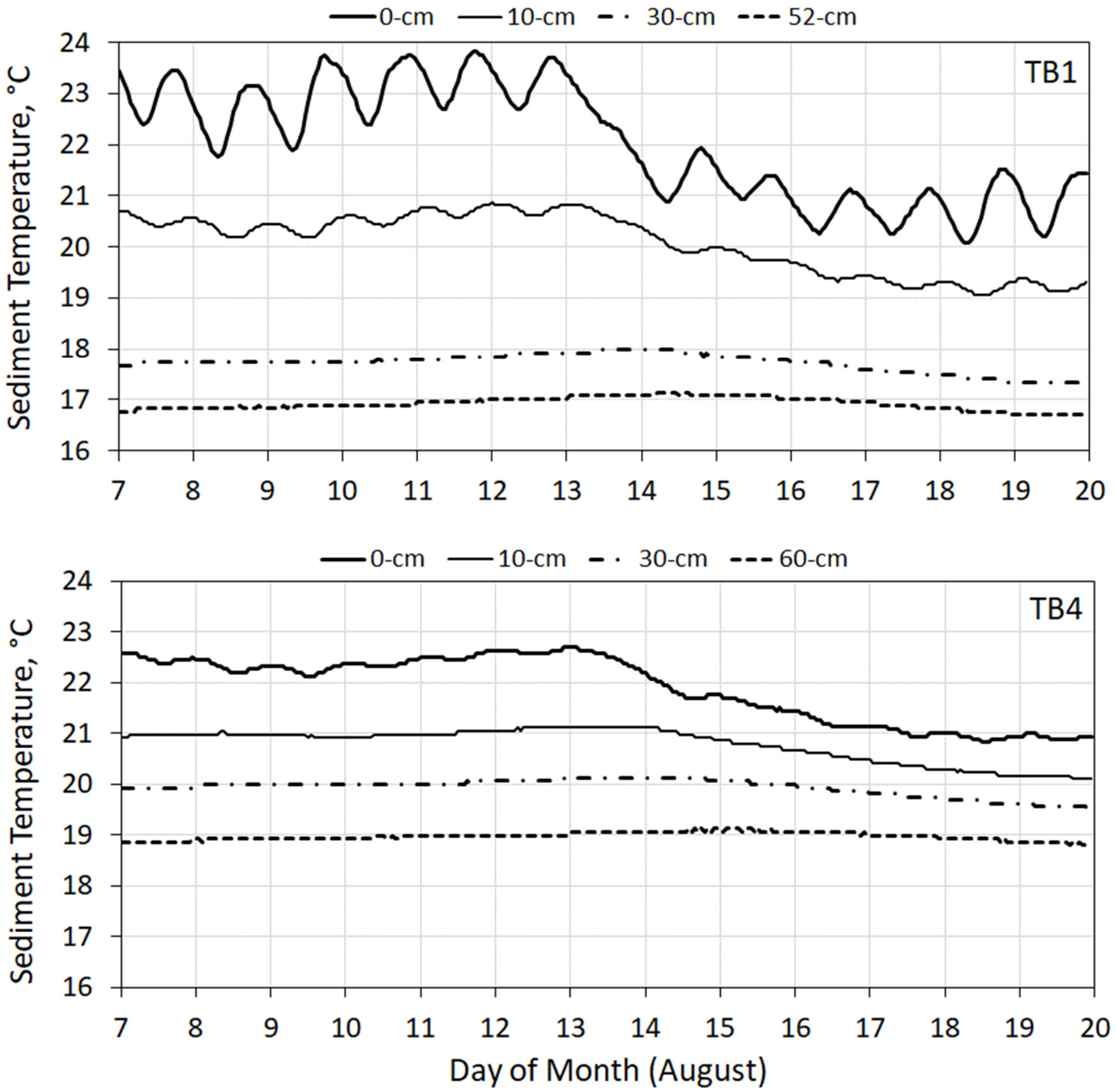

Figure S2. Sediment temperature trends at each depth for location TB1 (A) and TB4 (B) during the period of seepage flux analysis.

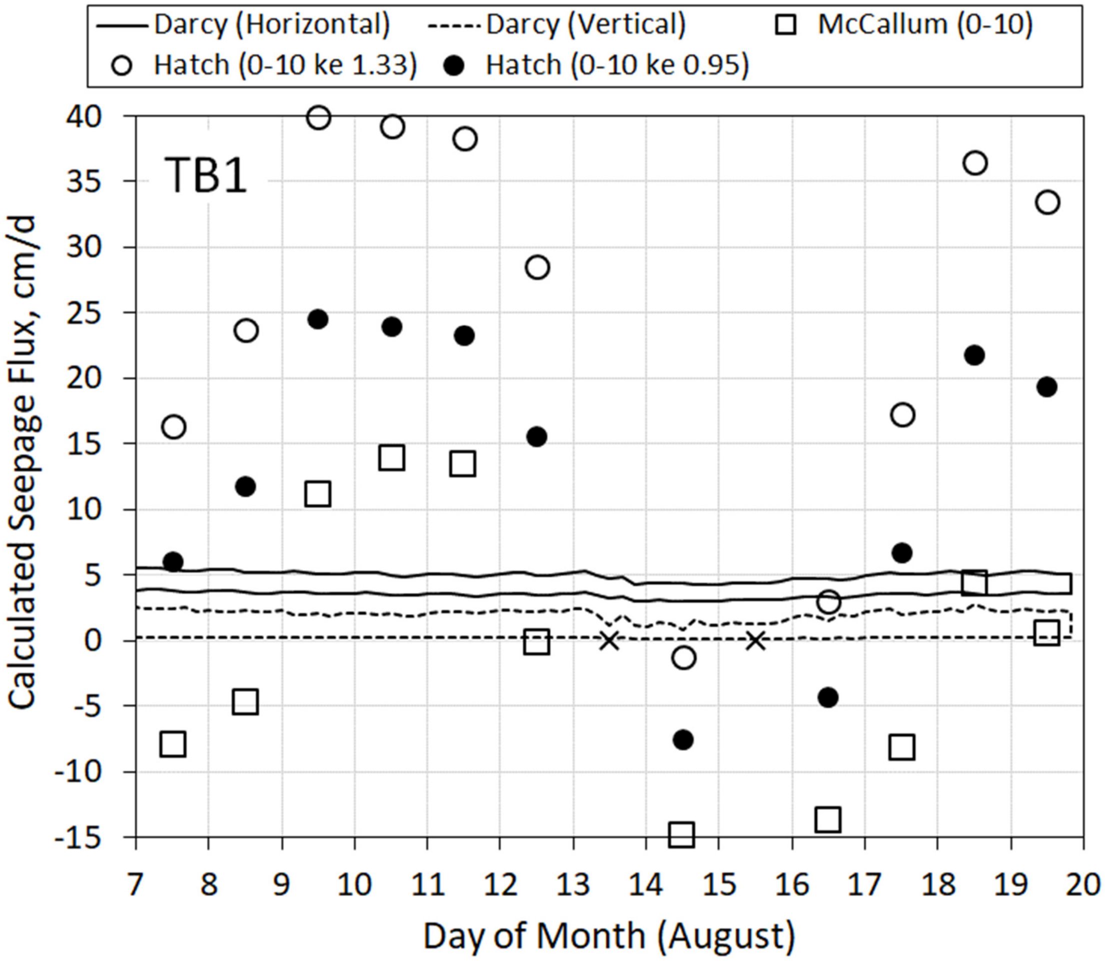

Figure S3. Trends in calculated seepage flux based on use of the Transient1DSE workbook for sediment temperature data from 0-cm and 10-cm depths at location TB1. Envelopes for the range of the horizontal (solid line) and vertical (dashed line) Darcy flux values were calculated using hydraulic gradient data for piezometer locations RSK12, RSK15, and PZ5 in combination with independent estimates of the range of hydraulic conductivity values for aquifer (20-31 m d^{-1}) and cove (0.3-3 m d^{-1}) sediments. Transient model input parameters: porosity 0.4, fluid heat capacity 4.16x106 J m^{-3} °C^{-1}, solid heat capacity 2.0016x10^6 J m^{-3} °C^{-1}, and dispersivity 0.001 m. Hatch model output is shown for input sediment thermal conductivity values of 1.33 W m^{-1} °C^{-1} (open circles) and 0.95 W m^{-1} °C^{-1} (filled black circles).

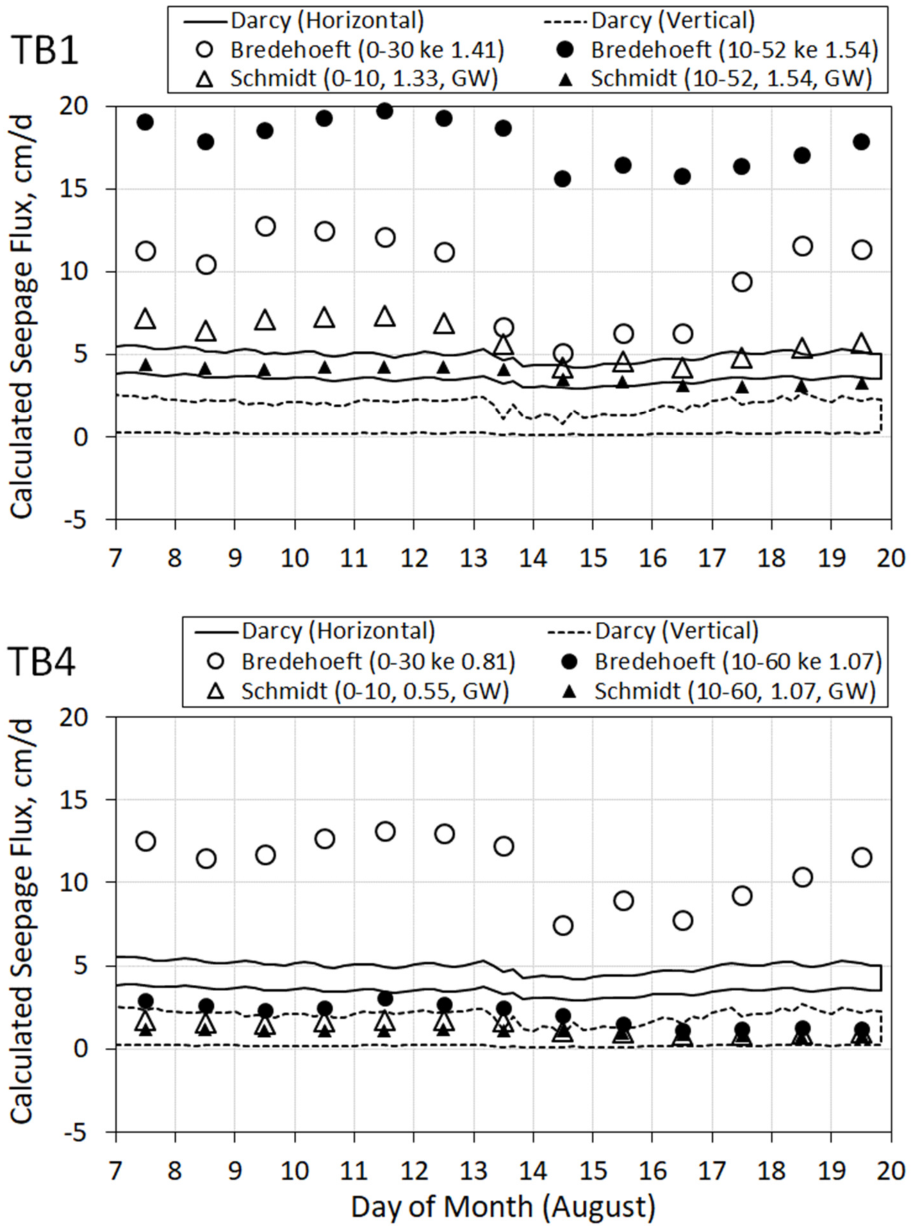

Figure S4. Trends in calculated seepage flux based on use of the SteadyState1DSE workbook for sediment temperature profile data at locations TB1 (top panel) and TB4 (bottom panel). Envelopes for the range of the horizontal (solid line) and vertical (dashed line) Darcy flux values were calculated using hydraulic gradient data for piezometer locations RSK12, RSK15, and PZ5 in combination with independent estimates of the range of hydraulic conductivity values for aquifer (20-31 m d^{-1}) and cove (0.3-3 m d^{-1}) sediments. Sediment thermal conductivity (W m-1 °C-1) values used for model input: TB1 (0-30 cm) 1.41, TB1 (10-52 cm) 1.54, TB4 (0-30 cm) 0.81, and TB4 (10-60 cm) 1.07. GW refers to groundwater temperature used in "Schmidt" model calculations (12.06 °C).

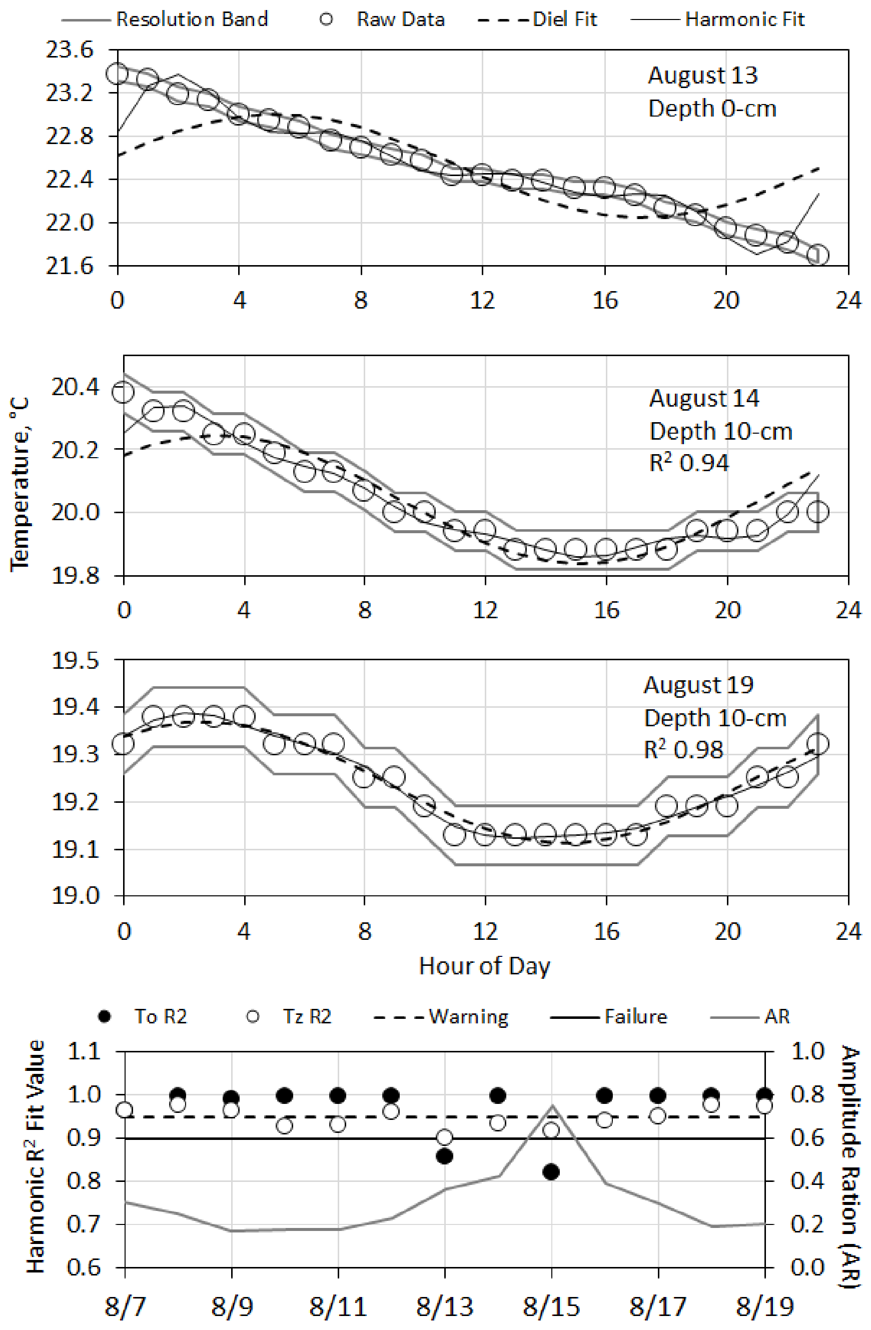

Figure S5. Comparison of the quality of harmonic regression fits to temperature data for three days during and after the rainfall event (top three panels). The light gray "resolution band" reflects the upper and lower bound on the ability of the iButton to resolve temperature changes. Bottom panel shows the quality of diel fits for temperature at 0-cm and 10-cm along with trend in amplitude ratio during the monitoring period. Warning and failure criteria are applied to combined harmonic fits.
